# Correction: Doxifluridine promotes host longevity through bacterial metabolism

**DOI:** 10.1371/journal.pgen.1012362

**Published:** 2026-11-03

**Authors:** Rui Wei, Yuling Peng, Yamei Luo, Xinyuan Wang, Zhenzhong Pan, Ran Zhou, Huan Yang, Zongyao Huang, Yaojia Liu, Lunzhi Dai, Yuan Wang, Yan Zhang

There are copy/paste errors in the S1 Fig, S3 Table and S1 Data. It can be viewed below.

## Supporting information

S1 Fig Pharmaceutical compounds were identified in the age-associated AS defects rescuing screen. (A) Schematic of the ret-1 splicing reporter. (B) Left, representative fluorescent images showing the ret-1 splicing reporter at Day 1 (D1) and Day 7 (D7). Right, quantification of GFP/mCherry ratio in (B). Each worm was measured by randomly selecting 8 regions in whole intestine. 6 worms were measured per group (Methods). n = 2 independent experiments. (C) Increased GFP/mCherry ratio in worms with 10 candidate drugs treatment compared to vehicle treatment. n = 2 independent experiments. (D) Fluorescent images of worms with lanolin, doxifluridine, 7213–0276 treatment. n = 2 independent experiments. (E-G) Lifespan curves of N2 worms treated with lanolin (E), doxifluridine (F), 7213–0276 (G). n = 2 independent experiments. (H) Mean lifespan extension percent of two experiment in worms treated with lanolin, doxifluridine, 7213–0276. (I) Fluorescent images of splicing reporter worms at Day 7 (D7) treated with different concentrations of FUDR. n = 2 independent experiments. (J) GFP/mCherry fluorescent intensity ratio in I. Around 10 worms were measured per group (Methods). n = 2 independent experiments. (K) Inhibition ratio of human hepatic cell line LO2 growth by different concentrations of doxifluridine and FUDR. Summary of three independent experiments, with each point representing one replicate in S1K Fig. n = 3 independent experiments. (L) Normalized ratio of GFP/mCherry in metformin, rapamycin and doxifluridine-treated worms. n = 2 independent experiments. The lifespan data illustrated in the figure corresponds to one repeat experiment. Error bars, SEM. *P < 0.05, **P < 0.01, ***P < 0.001, ****P < 0.0001. B, C, J, K, unpaired two-tailed Student’s t test. E-G, Log-rank (Mantel-Cox). L, one-way ANOVA. B, D, I, scale bars, 100 μm.
(TIF)

S3 Table Lifespan analysis. (XLSX)

S1 Data Source data. (XLSX)
