# Supplementary figures and images for: Doxifluridine promotes host longevity through bacterial metabolism
Source: PLoS Genet. 2025 Mar 31;21(3):e1011648. doi: 10.1371/journal.pgen.1011648 (PMC11977963; doi:10.1371/journal.pgen.1011648)

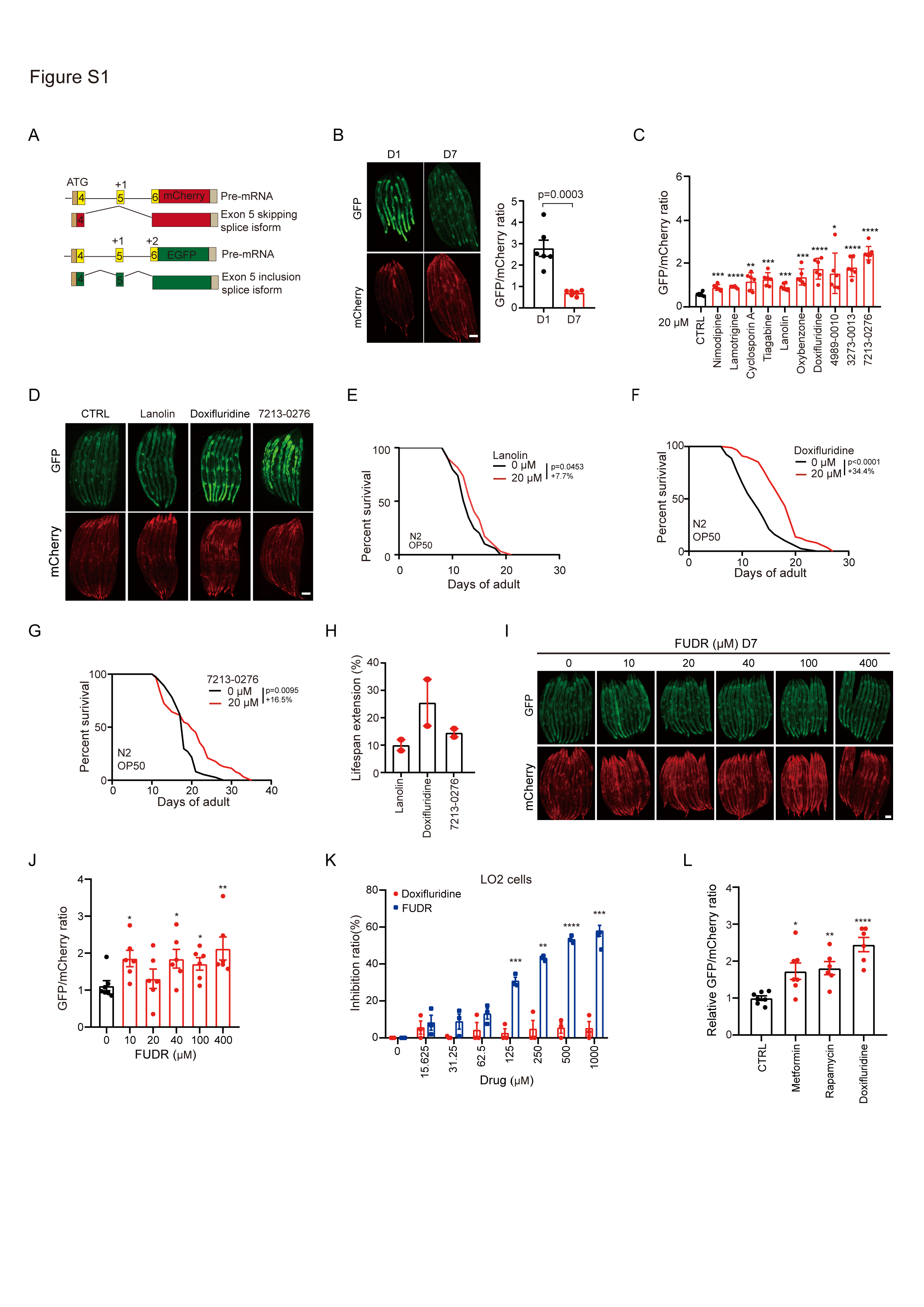

Supplement: S1 Fig — (A) Schematic of the ret-1 splicing reporter. (B) Left, representative fluorescent images showing the ret-1 splicing reporter at Day 1 (D1) and Day 7 (D7). Right, quantification of GFP/mCherry ratio in (B). Each worm was measured by randomly selecting 8 regions in whole intestine. 6 worms were measured per group (Methods). n=2 independent experiments. (C) Increased GFP/mCherry ratio in worms with 10 candidate drugs treatment compared to vehicle treatment. n=2 independent experiments. (D) Fluorescent images of worms with lanolin, doxifluridine, 7213–0276 treatment. n=2 independent experiments. (E-G) Lifespan curves of N2 worms treated with lanolin (E), doxifluridine (F), 7213–0276 (G). n=2 independent experiments. (H) Mean lifespan extension percent of two experiment in worms treated with lanolin, doxifluridine, 7213–0276. (I) Fluorescent images of splicing reporter worms at Day 7 (D7) treated with different concentrations of FUDR. n=2 independent experiments. (J) GFP/mCherry fluorescent intensity ratio in I. Around 10 worms were measured per group (Methods). n=2 independent experiments. (K) Inhibition ratio of human hepatic cell line LO2 growth by different concentrations of doxifluridine and FUDR. Summary of three independent experiments, with each point representing one replicate in S1K Fig. n=3 independent experiments. (L) Normalized ratio of GFP/mCherry in metformin, rapamycin and doxifluridine-treated worms. n=2 independent experiments. The lifespan data illustrated in the figure corresponds to one repeat experiment. Error bars, SEM. *P < 0.05, **P < 0.01, ***P < 0.001, ****P < 0.0001. B, C, J, K, unpaired two-tailed Student’s t test. E-G, Log-rank (Mantel-Cox). L, one-way ANOVA. B, D, I, scale bars, 100 μm. (TIF) [file pgen.1011648.s001.tif]

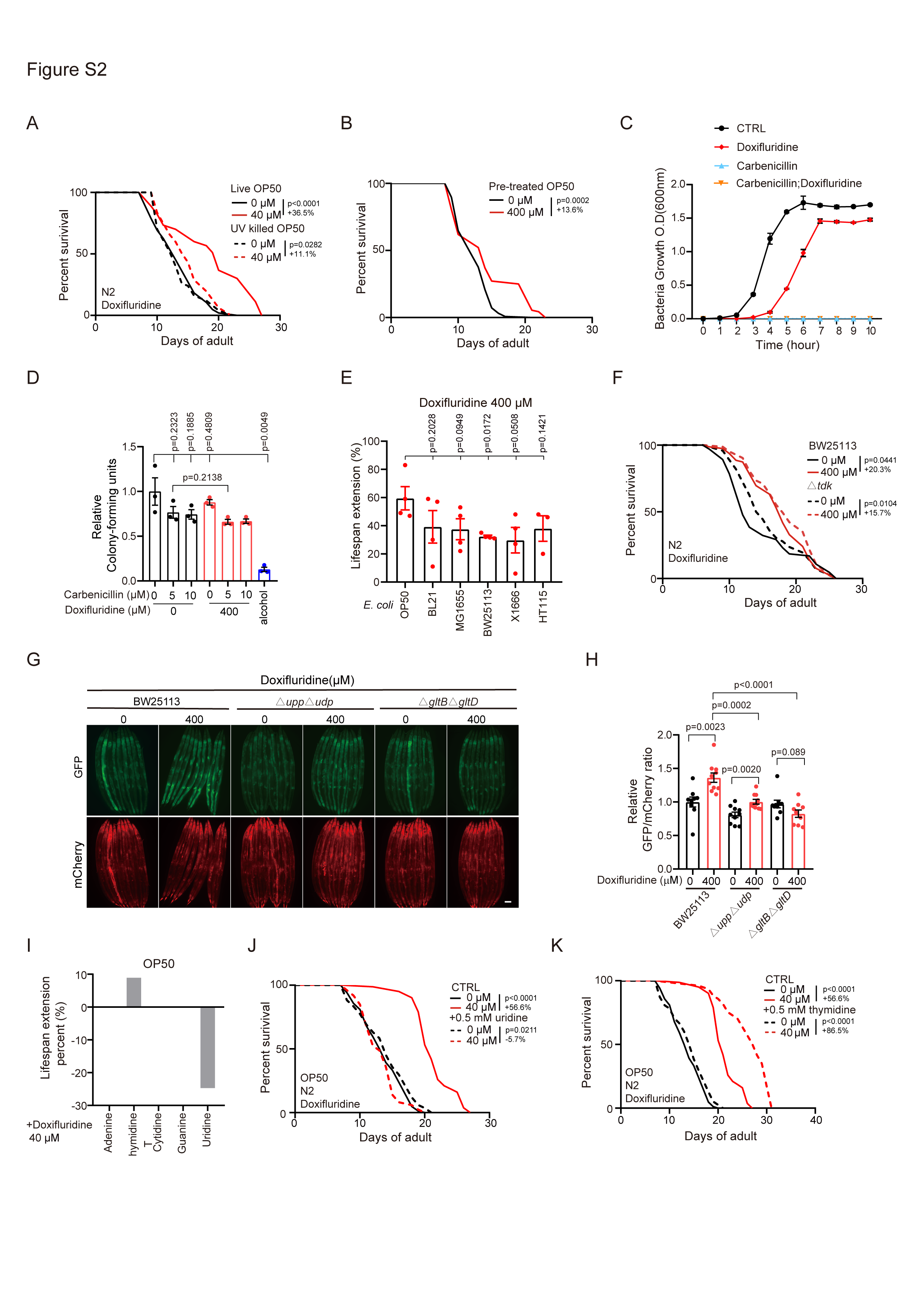

Supplement: S2 Fig — (A) Lifespan curves of worms treated with or without doxifluridine 40 μM in UV-killed bacteria. n=2 independent experiments. (B) Lifespan curves of worms in doxifluridine pretreated with OP50. n=2 independent experiments. (C) The growth curves of OP50 in doxifluridine (400 μM), carbenicillin (5 μM), and a blank control. Combination of three independent experiments. n=3 independent experiments. (D) Relative colony-forming units (CFUs) of OP50 bacteria after treatment with 0 μM, 5 μM, and 10 μM carbenicillin, with or without the addition of doxifluridine. n=2 independent experiments. (E) Mean lifespan extension of worms treated with doxifluridine (400 μM) in different laboratory bacteria strains. E. coli K-12 strains: BW25113, HT115, MG1655, X1666; E. coli B strains: OP50, BL21. (F) Lifespan curves of worms treated with or without doxifluridine 400 μM in BW25113 and BW25113 △tdk. n=2 independent experiments. (G) Fluorescent images of splicing reporter worms with or without doxfluridine 400 µM treatment in wildtype, ΔuppΔudp, ΔgltBΔgltD BW25113 bacteria. n=2 independent experiments. (H) GFP/mCherry fluorescent intensity ratio in G (9–10 worms were measured per group). n=2 independent experiments. (I) Relative lifespan extension percent in worms treated with doxifluridine 40 μM in OP50 adding with nucleosides (adenine, thymidine, cytidine, guanine and uridine) respectively, normalized to OP50 (CTRL) group. (J and K) Lifespan curves in worms treated with or without doxifluridine 40 μM in OP50 adding with uridine (J), thymidine (K) respectively. n=2 independent experiments. Lifespan curves of OP50 treated with and without doxifluridine 40 µM were shown twice in (J) and (K) for comparison. The lifespan data illustrated in the figure corresponds to one repeat experiment. Error bars, SEM. A, B, F, J, K, Log-rank (Mantel-Cox) test. D, E, H, unpaired two-tailed Student’s t test. G, scale bars, 100 μm. (TIF) [file pgen.1011648.s002.tif]

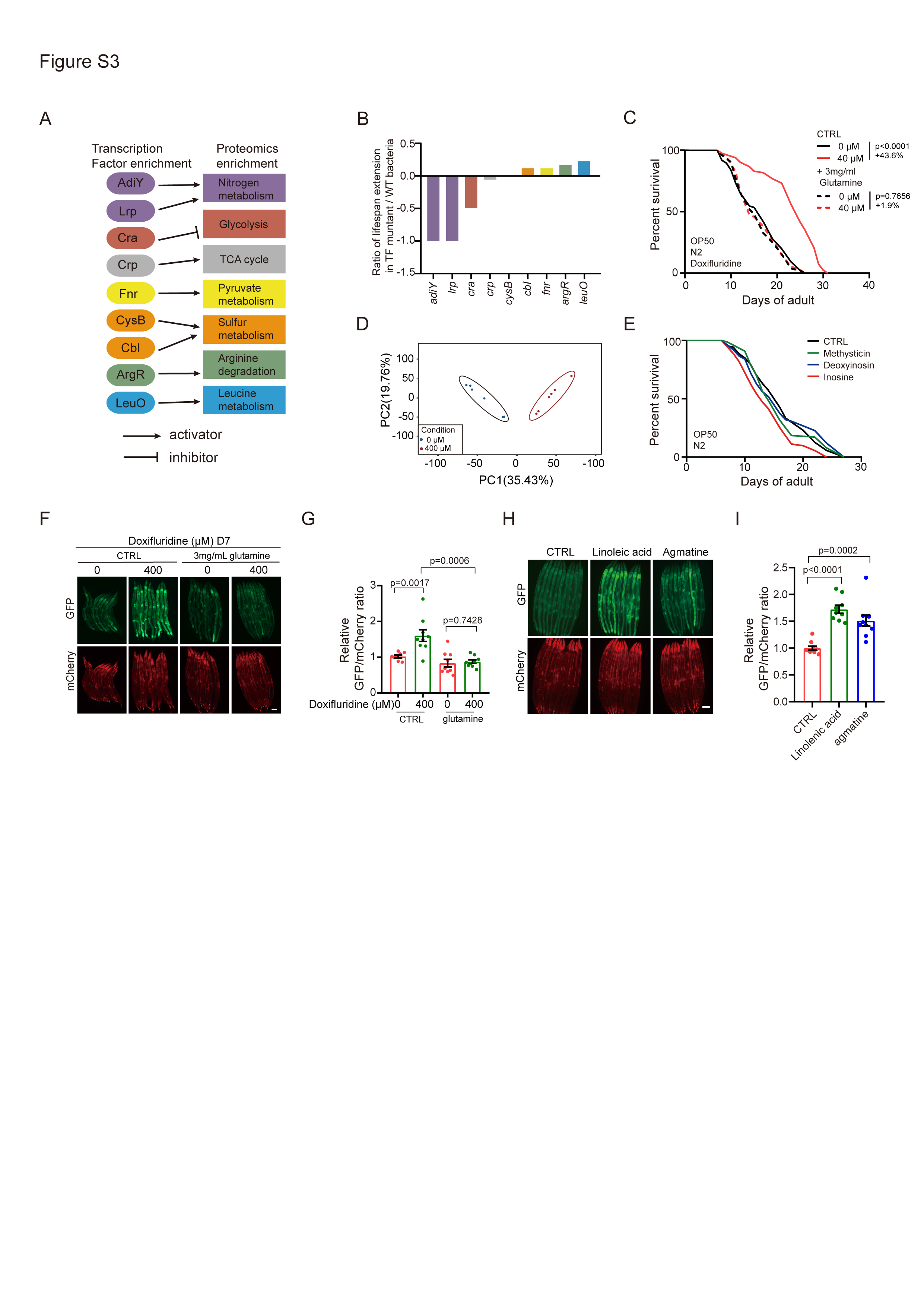

Supplement: S3 Fig — (A) Diagram displaying the RegulonDB transcription factors (TF) that regulate the metabolic pathway from proteomics data in E. coli OP50 treated with doxifluridine. (B) Relative lifespan extension of worms treated with doxifluridine in TF-mutant bacteria groups, normalized to BW25113 (WT) group. (C) Lifespan curves of worms treated with or without doxifluridine 40 μM in OP50 with or without glutamine supplementation. n=2 independent experiments. (D) PCA plot of E. coli OP50 metabolomics data showing effect of doxifluridine-treatment (400 μM) or vehicle-treatment (0 μM) in E. coli OP50. (E) Lifespan curves of worms treated with methysticin (91.15 μM), deoxyinosine (436 μM), inosine (913.35 μM) or vehicle. n=2 independent experiments. (F) Fluorescent images of splicing reporter worms with or without doxfluridine 400 µM treatment in OP50, OP50 with 3mg/mL glutamine. n=2 independent experiments. (G) Relative GFP/mCherry fluorescent intensity ratio in F (9–10 worms were measured per group). n=2 independent experiments. (H) Fluorescent images of splicing reporter worms treat with linoleic acid (75 µM) and agmatine (25 mM). n=2 independent experiments. (I) Relative GFP/mCherry fluorescent intensity ratio in H (9–10 worms were measured per group). n=2 independent experiments. The lifespan data shown in the figure is one of the two replicates. C, E, Log-rank (Mantel-Cox) test. G, I, unpaired two-tailed Student’s t test. F, H, scale bars, 100 μm (TIF) [file pgen.1011648.s003.tif]
