# Supplementary material for: Doxifluridine promotes host longevity through bacterial metabolism
Source: PLoS Genet. 2025 Mar 31;21(3):e1011648. doi: 10.1371/journal.pgen.1011648 (PMC11977963; doi:10.1371/journal.pgen.1011648)
Supplement: Attachment — Submitted filename: response to Reviewers ZY.pdf [file pgen.1011648.s015.pdf]

Response to Reviewers' Comments (PGENETICS-D-24-01277)

We would like to thank these reviewers for their thorough and high-quality reviews. Their critiques have greatly helped to improve the manuscript. Following their constructive suggestions, we have substantially revised our manuscript and figures. Below, we provide a point-by-point rebuttal to the issues they raised. The revised text is highlighted in yellow in the manuscript.

Reviewer #1: "*This manuscript describes an interesting phenomenon in which the nucleotide analog doxifluridine increases C. elegans lifespan. The authors show that live bacterial cultures with specific wild-type nucleotide metabolism enzymes are necessary for this extension. Although many questions remain about the specific links between drug administration and organismal phenotypes, the manuscript is for the most part well-supported and convincing.*"

Response: Thank you for your summary and positive comments.

Major comments:

1. "*It is important to explain more about the reporter system used for screening, in both the results, figure, and figure legend. What gene is being targeted? Which exon(s) of that gene? Is there something special about this alternative splicing that is coupled to lifespan? Or are all alternative splicing events likely to have similar patterns with aging and similar responses to drug administration?*"

Response: We appreciate your insightful comment. We have added a more detailed explanation of the reporter system in our revised manuscript, figure and figure legends (Revised Figure S1A, Line 105-109). The reporter strain, KH2235, expresses a pair of *ret-1* exon 5 reporter minigenes with differential frameshifts. GFP expression indicates the inclusion of exon 5, while mCherry expression indicates exon 5 skipping. These *ret-*

1 splicing events change with aging and are regulated by splicing factor 1 (SFA-1), which is required for lifespan extension.

Revised Figure S1A

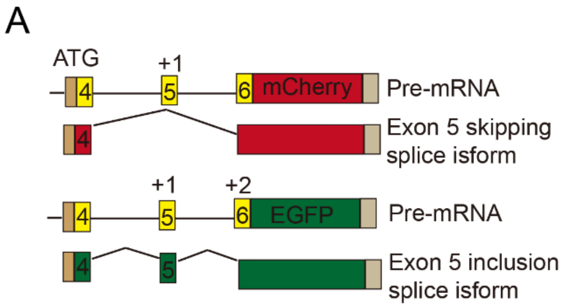

To investigate changes in alternative splicing events during aging and in response to drug administration, we conducted RNA-seq experiments on young worms (D1), aged worms (D9), aged worms treated with doxifluridine. We found that aged day 9 worms exhibited an increase in unannotated splice junctions and intron retention, indicating a rise in splicing noise with age. Doxifluridine treatment significantly reversed these alterations, restoring splicing patterns to levels comparable to D1 (Revised Figure 1D-F, Line 137-143). Additionally, we conducted Gene Ontology (GO) analysis on genes showing aging-related changes in alternative splicing that could be reversed by doxifluridine treatment. The enriched pathways revealed several critical cellular functions, including cytoskeleton organization, regulation of the cell cycle, chromatin organization, and more (Revised Figure 1G and 1H, Line 143-151).

Revised Figure 1D-F

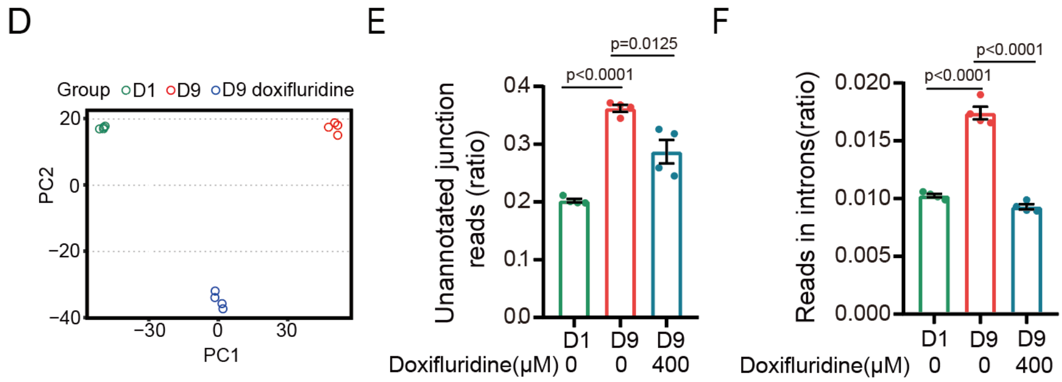

Revised Figure 1G and 1H

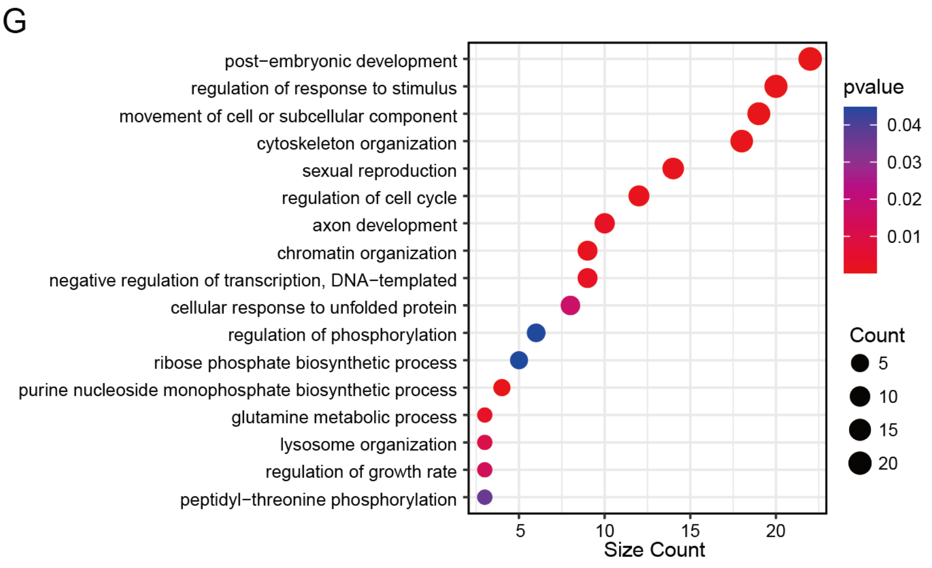

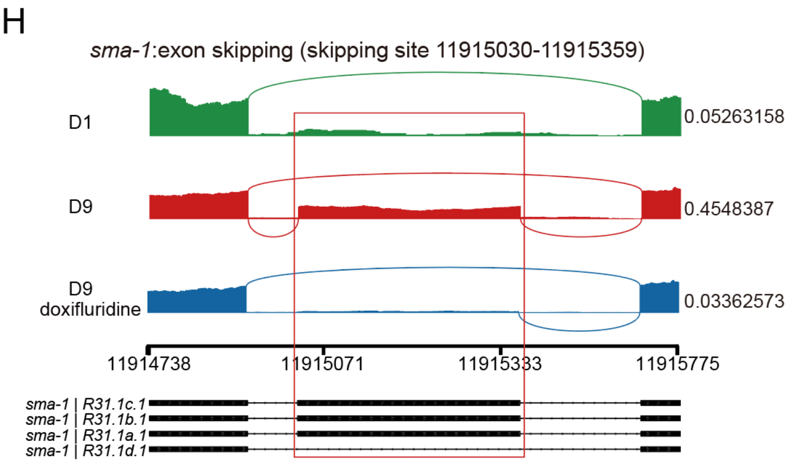

2. "*The authors claim in the abstract and intro that they "demonstrate its mechanism of action" by which doxifluridine increases C. elegans lifespan. I find this claim to be an overreach, as by no means do we leave with a clear molecular mechanistic pathway leading from doxifluridine to organismal lifespan. We have a few observations (live bacteria are required, certain bacterial genes are required), but much work would remain to support the claim to "demonstration of the mechanism of action.'*""

Response: We agree with you and have removed the description accordingly (Line 43).

3. "*Greater clarity and impact would be achieved if a clearer link could be made*

between the initial screened phenotype (splicing reporter) and the phenotype followed for the rest of the paper (lifespan). Are there any conditions in which these two phenotypes can be decoupled? Is one phenotype 'upstream' of the other? i.e. does mis-splicing lead to aging, does aging lead to mis-splicing, or are both phenotypes a consequence of some other upstream initiator? This would go a long way toward strengthening the mechanism of action claim above. Practical experiments that could partially address this question would be (a) live bacteria are required for the lifespan phenotype of doxifluridine; what about the splicing reporter phenotype? (b) same question about the ribonucleotide metabolism mutants; (c) same question but about glutamine (and other metabolite) supplementation. Additional experiments would be required for a full mechanistic explanation, but these would be a good (and easy) first step."

Response: We fully agree that further explanation is needed to establish the connection between the splicing phenotype and lifespan extension mediated by doxifluridine. In response to your suggestion, we conducted additional experiments. First, we examined changes in the splicing reporter phenotype in both live bacteria and heat-inactivated bacteria treated with doxifluridine. Our results indicate that live bacteria are necessary for the doxifluridine-induced splicing reporter phenotype (Revised Figure 2E and F, Line 179-183). Additionally, ribonucleotide metabolism mutant *ΔuppΔudp*, glutamate synthase mutant Δ*gltB*Δ*gltD* bacteria and glutamine supplementation, those of which block lifespan extension in doxifluridine-treated worms, also inhibited the doxifluridine-induced increase in the GFP/mCherry expression ratio (Revised Figure S2G and 2H, Line 240-245; S3F and 3G, Line 287-290). Furthermore, supplementation of the medium with downstream metabolites, such as linolenic acid, not only extended lifespan but also resulted in a higher GFP/mCherry expression ratio (Revised Figure S3H and I, Line 308-310). These findings suggest that doxifluridine-induced lifespan extension and the rescue of age-related alternative splicing are mechanistically linked.

Revised Figure 2E and F

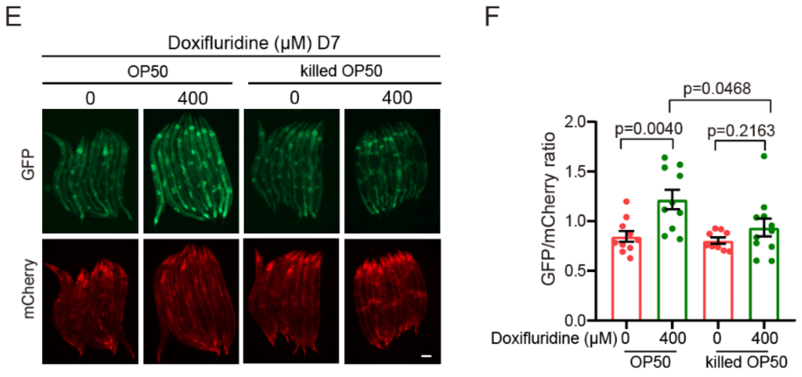

Revised Figure S2G and H, S3F and G

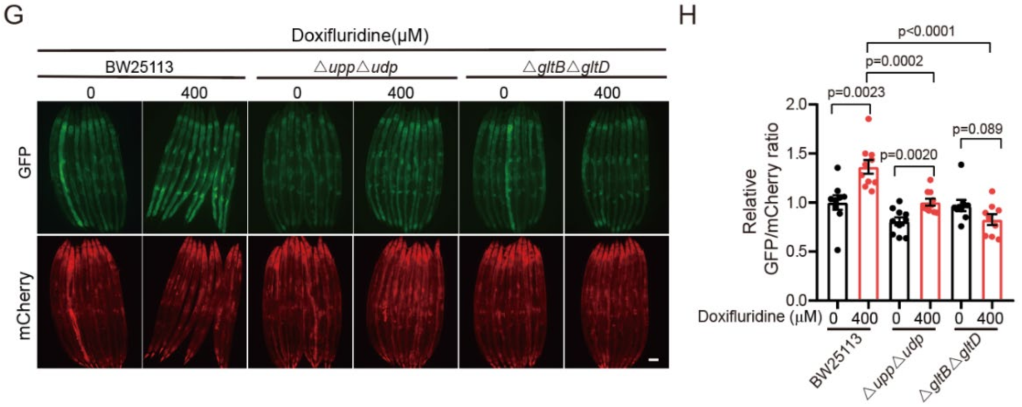

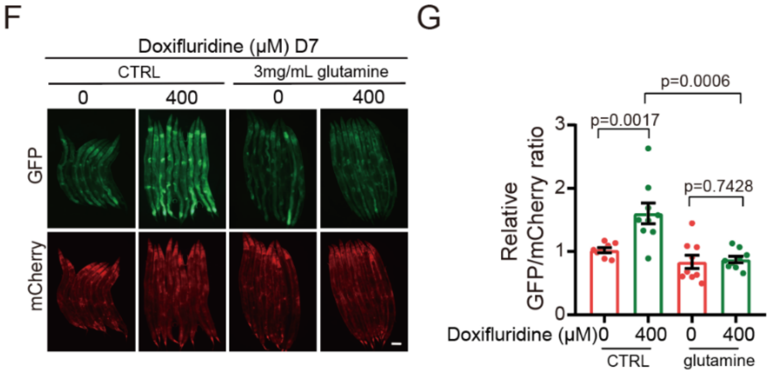

Revised Figure S3H and I

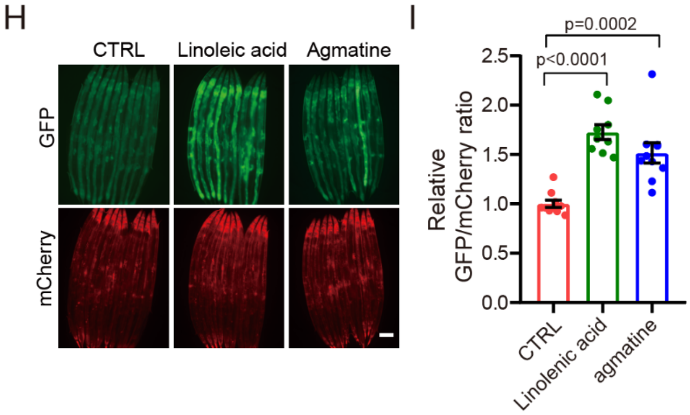

Minor comments:

1. "*The use of the word "consistently" is inconsistently and sometimes incorrectly used. Sometimes it is used correctly, in the sense of "invariably." But other times it is used incorrectly, in the sense of "this is consistent with our hypothesis." If the latter meaning is intended, a different phrase should be used. Otherwise the implication is that "these data are reproducible or invariable across trials," without showing any such supporting data. Lines 104, 178, 222 are examples of mis-use.*"

Response: We agree with you and have removed the misused words.

5. "*Line 62 claims that aging is driven by disrupted transcriptional and protein homeostasis. I'd recommend a more nuanced and well-supported claim, such as "…disrupted transcriptional and protein homeostasis contributes to aging…"*""

Response: We agree with you and have revised the sentence to: "Disrupted transcriptional and protein homeostasis contributes to aging, characterized by progressive functional decline leading to increased mortality." (Line 62-63).

Reviewer #2: In the article entitle 'Doxifluridine promotes host longevity through bacterial metabolism', the authors conducted a screen covering thousands of chemicals aiming at identifying potential aging-combating medication. The novelty of using the efficient nematode model and its medical implications are impressive. However, a few points should be fully addressed to be scientifically convincing enough, as listed below.

Response: Thank you for your summary and thoughtful critiques.

Major points:

1. "*The association between alternative splicing defects and aging does not automatically justify using florescence as a phenotypic readout of longevity, as*

evidenced by the low overlapping ratio (3/10, Figure 1A, Table S1) presented by this article. Either a more appropriate screening method should be adopted, or the interpretations thus the purpose/significance of this screen should be modified accordingly."

Response: Thank you for the constructive comment. We have added the interpretations of our purpose of this screen as "We aim to determine whether the drug not only rescues age-associated alternative splicing (AS) defects but also extends lifespan, as the AS changes induced by the drug may not necessarily be linked to lifespan extension." in revised manuscript (Line 119-121).

2. "*The lifespan of C. elegans observed in this study is substantially shorter than most reported in the principle. The N2 worms feeding on live OP50 at 20C typically exhibit an average lifespan of 17-19 days (e.g. PMID：17081160；PMID：23415229). Plus, the data show that the population starts to decease at Day 5-6 (e.g. Figure 1D), even well before complete cessation of reproduction, which is so unlikely that it makes all the longevity information across the article questionable. Possible reasons for this aberrant lifespan include: 1) involuntary artificial selection on the N2 population; 2) rough manipulation of the experimental animals that results in physical injuries; 3) contamination of the cultivating medium by harmful environmental germs. The authors didn't provide the detailed information on animal maintenance or the sterility techniques used during screens and validation, making estimation on the likelihood of above reasons improbable at this point. Anyway, this lifespan issue should be solved to ensure that any conclusions obtained indeed relevant to the aging processes.*"

Response: Thank you for your suggestions. We carefully monitored the animal maintenance conditions and used worms that met our criteria, excluding the three potential issues mentioned above. If any of these issues occurred, we repeated the experiments. It is well-established that the lifespan of N2 worms fed with live OP50 at

20°C can vary across different studies. For instance, some studies report an average lifespan of around 12 days (Hahn A et al., Cell Metab. 2024. PMID: 39173633; Traa A et al., Aging Cell. 2024. PMID: 38953684). However, we observed that some worms dying during the reproduction phase may be due to worm bagging defects. To address this, we reanalyzed the lifespan data by focusing on worms that died after the reproduction phase. Additionally, we have included further details on animal maintenance, sterility techniques, and experimental protocols used during the screening and validation processes in the Methods section (Line 360-362, 373-376, 428-435).

3. "*It is well acknowledged that killing or inactivation of bacteria is sufficient to lengthen lifespan of C. elegans. Before concluding that doxifluridine is a longevity-promoting molecule, the authors should rule out the possibility that this compound directly limits survival of symbiotic E. coli— in other words, it is simply an antibiotic. Monitoring influences of high-concentration doxifluridine on the bacterial growth curves would be helpful.*"

Response: Thank you for your insightful suggestions. Following your recommendation, we tested bacterial growth curves under 400 μM doxifluridine. We found that doxifluridine exhibited moderate inhibition of bacterial proliferation (Revised Figure S2C). We tested whether the effect of doxifluridine on bacteria is bactericidal or bacteriostatic. We subcultured the bacteria from doxifluridine-treated plates and found that *E. coli* show no significant reduction in colony-forming units (Revised Figure 2G, Figure S2D). Additionally, the morphology and quantity of bacteria treated with doxifluridine were comparable to those of untreated bacteria (Revised Figure 2H). These results demonstrate that doxifluridine does not exhibit bactericidal activity. We then investigated whether the lifespan-extending effect of the doxifluridine depend on its antibiotic effect. To test this, we assessed the drug's effect in the presence of carbenicillin, a classic antibiotic that inhibits bacterial proliferation. While carbenicillin

suppressed bacterial growth more effectively than doxifluridine (Revised Figure S2C), it resulted in less lifespan extension compared to doxifluridine (Revised Figure 2I). Importantly, doxifluridine still extended worm lifespan even under carbenicillin-induced bacterial inhibition, indicating that its lifespan-extending effect does not rely on its antibacterial activity (Revised Figure 2I) (Line 185-200).

Revised Figure S2C, S2D, Revised Figure 2G

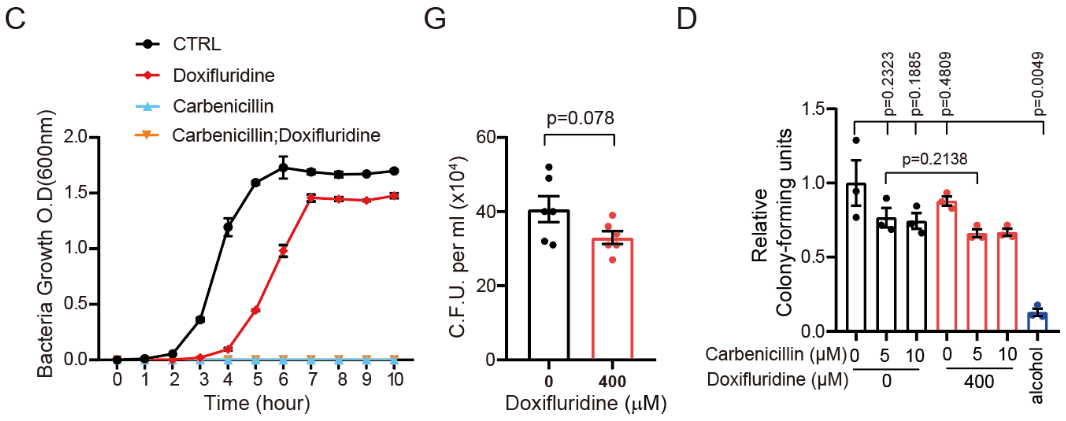

Revised Figure 2H and I

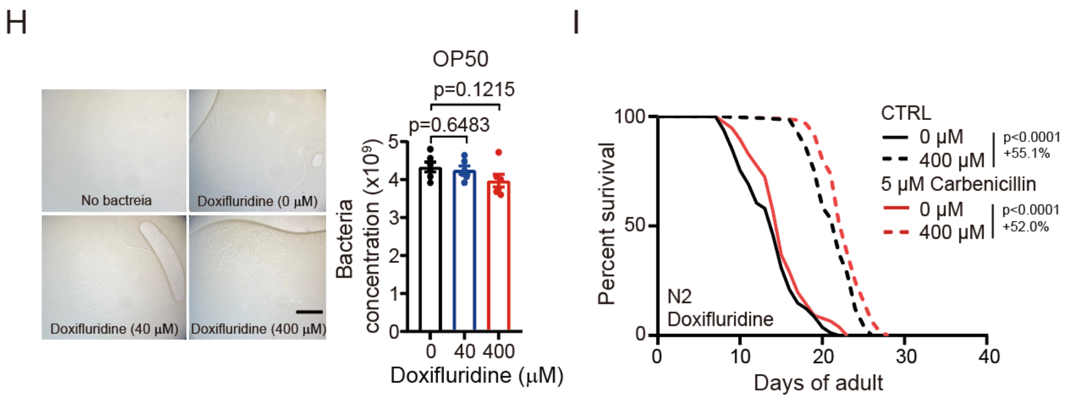

Minor points:

1. "*The authors should provide information on criteria or methods of judging solubility of the drugs. It is even not clear whether doxifluridine is water or DMSO controlled.*"

Response: We sincerely apologize for any confusion. We have provided detailed information on the dissolution methods for doxifluridine, uridine, thymidine, glutamine,

and other compounds mentioned in the manuscript. These details have been added to the Methods section (Line 441-452). The doxifluridine used in our experiments is water-soluble, with a maximum solubility in water of 182.8 mM.

2. "*It should be clarified how the progeny of experimental animals were excluded from analyses during the screen.*"

Response: Thank you for your suggestions. During the screening process, we transferred the original worms to fresh, bacteria-seeded NGM plates daily or every two days. This approach effectively excluded progeny from the analyses, ensuring that the results were not confounded by the presence of offspring. We have included a detailed description of this procedure in the revised Methods section (Line 372-375).

3. "*The rationals behind bacterial strain selection (e.g. OP50 vs. BW25113) at each point should be explained.*"

Response: Thanks for your suggestions. OP50 is the standard bacterial strain used for routine maintenance of *C. elegans* in most experiments, and we initially used OP50 in our preliminary studies. However, we discovered that the effects of doxifluridine depend on bacterial metabolism. To identify the bacterial metabolic enzymes involved in the efficacy of doxifluridine, we utilized a commercial library of BW25113 mutant strains. We have clarified this rationale and selection process in the revised manuscript (Line 227-228, 270)

Reviewer #3: "*This interesting and well-written paper reports the identification of the compound doxifluridine as a potential avenue for rescuing age-associated alternative splicing defects and extending lifespan. The authors use a C. elegans screening platform to screen a large number of compounds and characterize their top hit doxifluridine. They show that doxifluridine has a strong efficacy at preventing age-associated AS decline in C. elegans, increasing resistance to multiple types of stress,*

improving age-related intestinal phenotypes, and extending lifespan. They further find that the lifespan extension by doxifluridine requires bacterial metabolism and identify several specific bacterial pathways including ribonucleotide metabolism, nitrogen metabolism, and agamatine synthesis that are required. I have some concerns regarding the number of replicates for certain experiments and certain information that is missing from the Methods, but overall the authors' conclusions are supported by the data presented. The study is well conceived and likely to be of interest to the aging research community, especially because doxifluridine has already been entered into phase II and III clinical trials in elderly populations."

Response: Thank you for your summary and positive comments. Regarding the concerns raised about the number of replicates for certain experiments, we have carefully reviewed the relevant data and, where necessary, have increased the number of replicates to ensure reproducible results. We hope these revisions address your concerns and enhance the clarity and reproducibility of our study.

Major concerns:

1. "*My most substantive concern is that a few of the experiments appear to have been performed only a single time and were not replicated. In particular for Figure 1C, the figure legend states that "6-8 worms were measured per group" and references the Methods, but I could not find any information in Methods as to whether this experiment had been performed a single time with 6-8 worms, or had been repeated with independent biological replicates. This also applies to the muscle mitochondrial analysis (Figure 1H-I) and the intestinal width analysis (Figure 1J), which also do not list in the Methods or in the figure legend if it was replicated. In addition for Figure 1J the Methods states that 30 worms were imaged but the figure legend states that n=16. If these experiments have been replicated, that information should be provided in the Methods and in the figure legends. If they have not been, then the experiments should be repeated (I would recommend at least twice more, given the very low n) to ensure*

that the results are reproducible."

Response: We appreciate the opportunity to address your concerns and apologize for any confusion. To clarify, all experiments, including those in revised Figures 1C, 1I-L, 1N, and 1P, were conducted with at least two independent biological replicates. We have included the replicate data in the original data source table and specified the number of repetitions in the revised Methods section (Line 475,486,400-401) and figure legends. In revised Figure 1P (originally Figure 1J), we have clarified in the Methods that more than 16 worms were used for the intestine atrophy analysis (Line 472).

2. "*My other major concern is a lack of detail in the Methods sections for the drug screen and for the microscopy and image analysis. For the drug screen, it is not clear which method was used to synchronize the nematodes. The reporter analysis was also not conducted until D7 of adulthood after plating the worms as D1 adults, and it is not clear how the authors differentiated between the originally plated worms and progeny after 7 days or if sterility was induced somehow. In the microscopy section, the only details provided for the GFP/mCherry quantification are "Images were analyzed in ImageJ" (line 325). Additional detail is needed here including how ROIs for quantification were selected, whether any background correction was applied, and whether mean pixel intensity or integrated density was used for calculating fluorescence.*"

Response: We apologize for any confusion on the methods sections. We have added details in the Methods sections for the drug screen and for the microscopy and image analysis (Methods, line 372-376, 390-401). In the drug screen, nematodes were synchronized using bleach buffer (1.6M NaOH, 12%NaClO). 20-30 synchronized young (D1) adults were plated per well and incubated at 20 °C. Worms were transferred to fresh drug screen plates daily to separate from their progeny. In the microscopy section, the fluorescence intensity of each nematode is measured by randomly selecting eight regions (ROIs, 8×60 pixel×60 pixel) within the intestine and calculating the

average grayscale value. Subsequently, subtract the average fluorescence intensity of the background from the average fluorescence intensity of the intestine to correct for the background, obtaining the mean pixel intensity of the nematode.

Minor concerns:

1. "*The paper would be strengthened significantly if the authors could show a connection between the rescue of age-associated AS defects by doxifluridine and the effects of doxifluiridine on lifespan, for instance by showing that the downstream bacterial pathways they identified as required for the doxifluridine-mediated LS extension were also required for the effect on AS. As it stands, it is possible that the lifespan extension by doxifluridine and the rescue of age-associated decline in AS are via completely separate mechanisms. However, since the authors are not making the claim in this paper that it is the drug's rescue of age-associated AS defects that leads to the lifespan extension, this is merely a suggestion. They may wish to save this topic to be explored in future studies.*"

Response: Thank you for the thoughtful suggestions. We conducted additional experiments, which suggest that doxifluridine-induced lifespan extension and the rescue of age-related alternative splicing are mechanistically linked (See also Response to Reviewer 1, Major Concern 3).

2. "*For Figure 1E and 1F I was able to confirm from Table S2 that these experiments were repeated twice – however, this information should also be available in the relevant Methods sections and/or in the figure legends, which it currently is not.*"

Response: Thank you for the suggestions. We have added the number of repeated experiments in the figure legends and methods (Line 434).

3. "*Although the Methods and/or figure legends state that the lifespan experiments were*

repeated twice, it is unclear for all of the lifespan figures whether the data shown in the figure is one of the two replicates, or both combined. This should be clarified in the figure legends."

Response: We apologize for any confusion. The lifespan shown in the figure represents one of the two independent biological replicates, and we have clarified this in the figure legends.

4. "*In the Figure 2 legend it says "Error bars, SEM" but there are no error bars shown in this figure.*"

Response: We have removed the statement "Error bars, SEM" from the Figure 2 legend. Thank you for pointing this out.

5. "*In Figure 2, the authors are using the same controls in both Figure 2A and 2B, but in Table S2 it says that it is 2B and 3A that are the same.*"

Response: We apologize for the confusion. We have corrected this statement in revised Table S3.

6. "*In Figure 3, it is hard to tell which pairs of lifespans are statistically different from each other as p values are not provided in the figure or in the legend. This is also true in the text where they do give the p values (lines 173-175), but it is not entirely clear from the text which comparisons are being made to produce those values.*"

Response: We apologize for the confusion. We have also included the p-values in the revised figures and provided the comparisons in the text.
